# Novel Nucleoside Analogues as Effective Antiviral Agents for Zika Virus Infections

**DOI:** 10.3390/molecules25204813

**Published:** 2020-10-20

**Authors:** Marcella Bassetto, Cecilia M. Cima, Mattia Basso, Martina Salerno, Frank Schwarze, Daniela Friese, Joachim J. Bugert, Andrea Brancale

**Affiliations:** 1 Department of Chemistry, Swansea University, Swansea SA2 8PP, UK; marcella.bassetto@swansea.ac.uk; 2 School of Pharmacy and Pharmaceutical Sciences, Cardiff University, Cardiff CF10 3NB, UK; cimacm@cardiff.ac.uk (C.M.C.); bassomattia01@gmail.com (M.B.); m.salerno@bangor.ac.uk (M.S.); 3 Institut für Mikrobiologie der Bundeswehr, 80937 Munich, Germany; Frank1Schwarze@bundeswehr.org (F.S.); danielafriese@bundeswehr.org (D.F.); joachim1bugert@bundeswehr.org (J.J.B.)

**Keywords:** antiviral agents, aryloxyphosphoramidate prodrugs, Zika virus, in vitro screening

## Abstract

Previously considered a neglected *flavivirus*, Zika virus has recently emerged as a public health concern due to its ability to spread rapidly and cause severe neurological disorders, such as microcephaly in newborn babies from infected mothers, and Guillain-Barré syndrome in adults. Despite extensive efforts towards the identification of effective therapies, specific antivirals are still not available. As part of ongoing medicinal chemistry studies to identify new antiviral agents, we screened against Zika virus replication in vitro in a targeted internal library of small-molecule agents, comprising both nucleoside and non-nucleoside agents. Among the compounds evaluated, novel aryloxyphosphoramidate prodrugs of the nucleosides 2′-C-methyl-adenosine, 2-CMA, and 7-deaza-2′C-methyl-adenosine, 7-DMA, were found to significantly inhibit the virus-induced cytopathic effect in multiple relevant cell lines. In addition, one of these prodrugs exhibits a synergistic antiviral effect against Zika virus when applied in combination with an indirect antiviral agent, a l-dideoxy bicyclic pyrimidine nucleoside analogue, which potently inhibits vaccinia and measles viruses in vitro by targeting a host pathway. Our findings provide a solid basis for further development of an antiviral therapy for Zika virus infections, possibly exploiting a dual approach combining two different agents, one targeting the viral polymerase (direct-acting antiviral), the second targeting a host-directed autophagy mechanism.

## 1. Introduction

Zika virus (ZIKV) belongs to the *Flavivirus* genus of the *Flaviviridae* family; it is closely related to other arthropod-borne viruses of the same genus, such as Dengue (DENV), Yellow Fever (YFV), and West Nile (WNV) viruses. First serologically surveyed in Uganda in 1952 [1], it was found in West Africa in 1954 and then spread to Indonesia, Micronesia, Thailand, the Philippines, French Polynesia, and Easter Island-South Pacific [2]. After a first report in Brazil in May 2015, ZIKV has spread rapidly in several countries across the Americas, causing an epidemic which affected more than 850,000 patients [3], and leading the World Health Organisation to declare it a public health emergency of international concern in 2016 [4]. ZIKV is transmitted to humans by mosquitos of the *Aedes* species, mainly *Aedes aegypti* and *Aedes albopictus* [5]. The number of new cases has lately diminished, but the continuous increase in urbanisation and intercontinental travel, and the current climate changes, which are predicted to lead to the adaptation of the main vector *Aedes aegypti* to new, expanded geographic areas, pose the threat of future outbreaks [6]. ZIKV can also be transmitted through the sexual route, blood transfusions, and the maternal–foetal route [7].

Although usually associated with a self-limiting and mild illness, either asymptomatic or characterised by fever, skin rashes, conjunctivitis, headache, muscle and joint pain, during the recent large outbreaks in French Polynesia and Brazil, different congenital malformations and an enhanced risk of foetal loss have emerged as associated with ZIKV infections during pregnancy [8]. Infections with ZIKV not only cause foetal abnormalities and congenital microcephaly [9], but also a number of neurological complications in adults, such as the Guillain Barré syndrome [8]. This alarming evidence has prompted extensive efforts towards the development of both vaccines and specific therapeutic measures [10], but to date, both options are still unavailable for human use, limiting the treatment of infections to supporting care [11]. As a consequence, the identification of effective antiviral options is highly needed and greatly significant to people’s health worldwide.

The ZIKV replication cycle is closely related to that of other flaviviruses [12], suggesting the potential for antiflaviral agents to display therapeutic activity also in the case of Zika virus. In this context, antiviral nucleoside analogues are a particularly interesting class of compounds, as they currently represent the most important group of antiviral agents, with roughly 50% of all antiviral drugs on the market belonging to this category [13,14]. With few exceptions, these agents exert their mode of action at the viral polymerase level in their triphosphate form. Different prodrug approaches have been developed to aid the metabolic activation of parent nucleosides into their active form, with the McGuigan aryloxyphosphoramidate prodrug (ProTide) technology being the most effective currently available, exploited, among others, in the blockbuster anti-HCV drug sofosbuvir [15]. To date, different nucleoside analogues have shown antiviral effects against ZIKV replication in vitro and in vivo [16], including sofosbuvir itself [17].

In this study, we focused on the in vitro evaluation, using multiple cellular systems relevant to ZIKV replication, of a targeted small library of potential anti-ZIKV agents, focusing on diverse compounds developed in our medicinal chemistry group as potential antivirals for different viruses, including both nucleoside analogues and non-nucleoside small molecules. Within the compounds evaluated in our assays, novel aryloxyphosphoramidate prodrugs of the nucleoside analogues 2′-CMA and 7-DMA were identified as inhibitors of ZIKV-induced cytopathic effect in multiple screening systems, with antiviral EC_50_ values in the low micromolar range. In addition, one of these prodrugs also displays a synergistic antiviral effect when applied in combination with a previously reported antiviral *L*-nucleoside analogue, providing a solid basis for the further development of these agents into a viable therapeutic option to treat ZIKV infections.

## 2. Results and Discussion

### 2.1. Compound Selection

With the aim to identify novel inhibitors of ZIKV-induced cytopathic effect in cells, we decided to create a focused small library of in-house compounds, prioritising those ones which we had identified, or which we are currently developing, as potential antivirals for related *flaviviruses*. In particular, we decided to focus our efforts on five structural families in total. Two of these are non-nucleosides, as summarised in Table 1. First, we included 13 diverse structural analogues of **1a** (**1a**–**1o**): these compounds have been previously identified in our group as potent inhibitors of the replication of hepatitis C virus, which is also a *flavivirus* [18]. In addition, we screened a second series of 11 non-nucleoside compounds, **2a**–**2m**: **2a** has been previously reported as a potent inhibitor of ZIKV replication in Vero cells, with efficacy in reducing ZIKV viremia in vivo in an immunocompetent mouse model, and a mechanism of action pointing towards inhibition of the viral RNA-dependent RNA polymerase (RdRp) [19]. Different structural analogues of **2a**, among which we selected **2b**–**2m**, have been recently explored in our research group for their inhibition of human norovirus (hNoV) RdRp, and their ability to interfere with hNoV replication in a replicon system [20].

**Figure 1:**
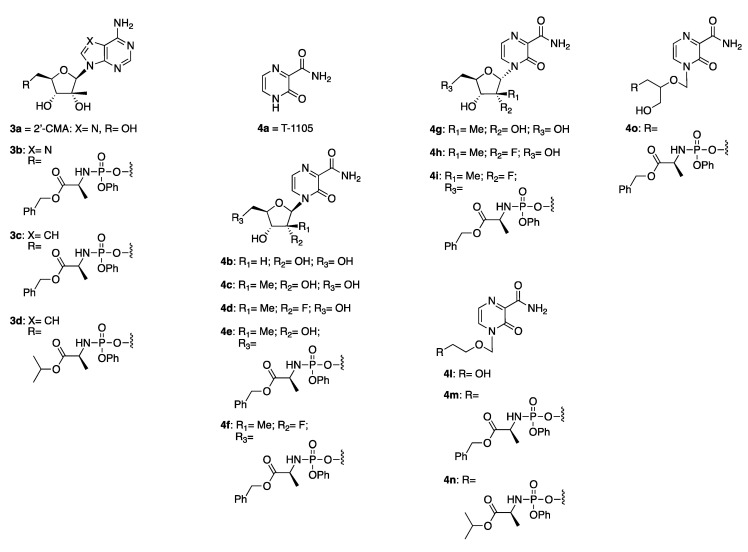
Chemical structures of nucleoside analogues **3a**–**3d** and **4a**–**4o**. The parent compounds **3a** and **4a** have been previously found to inhibit ZIKV replication in cell-based assays.

In parallel, we also tested three diverse structural families of nucleoside analogues. Figure 1 summarises the first two of these families.

Due to the anti-ZIKV activity previously found for 2′-CMA (**3a**) and 7-DMA [21,22], we prepared a small family of novel aryloxyphosphoramidate prodrugs of these two nucleosides (**3b**–**3d**) to evaluate the effect of the application of this prodrug technology [23] on the activity of the parent compounds. In addition, we prepared a series of nucleoside analogues (**4b**–**4o**) of the broad-spectrum antiviral agent T-1105 (**4a**), which has also been reported to inhibit ZIKV replication in cell-based assays, along with its close analogue T-705 (favipiravir), approved in Japan to treat influenza virus infections [24]. As T-1105 and T-705 exert their antiviral activity by inhibiting the viral polymerase after metabolic conversion into their ribofuranosyl 5′-triphosphate forms [24], a small series of ribofuranose analogues of T-1105 and their aryloxyphosphoramidate prodrugs was designed (**4c**–**4i**) to modify the sugar part of the nucleoside with the insertion of a 2′-C-methyl substituent, as present in 2′-CMA and 7-DMA, and to introduce the same replacement of the 2′-hydroxy group with a fluorine atom, as present in sofosbuvir. Since the corresponding α-anomers of nucleosides **4c** and **4d** were obtained during their synthetic preparation (**4g**, **4h**), these were also included in our assays, along with one aryloxyphosphoramidate prodrug of **4h** (**4i**), and the unmodified ribofuranosyl analogue **4b**. Finally, as our group is also exploring the antiviral potential of acyclic analogues of T-1105 (unpublished data), acyclic compound **4l** was also considered, along with acyclic aryloxyphosphoramidate prodrugs **4m**, **4n**, and **4o**.

**Figure 2:**
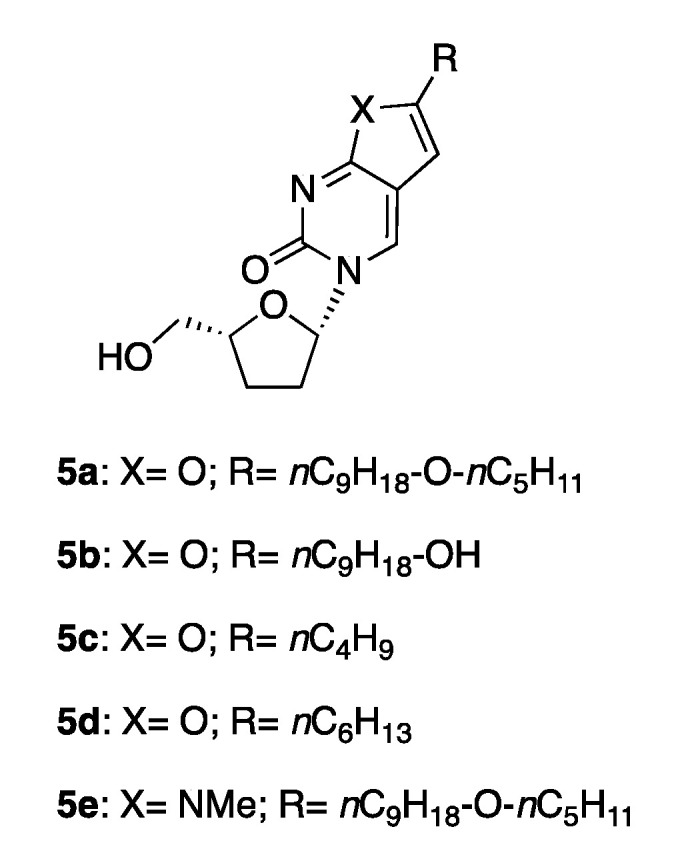
Chemical structures of nucleoside analogues **5a**–**5e**. These molecules are currently being investigated in our group for their potential antiviral activity against different *flaviviruses*; the scaffold of **5a** has been previously reported for its indirect antiviral properties against vaccinia and measles viruses.

Finally, an additional small series of nucleoside-based compounds, **5a**–**5e**, was chosen for evaluation against ZIKV-induced cytopathic effect in our systems (Figure 2).

This small series of l-dideoxy bicyclic pyrimidine nucleoside analogues **5a**–**5e** was included in our evaluation as among them; **5a** has been previously found to potently inhibit the replication of both measles and vaccinia viruses in vitro through an indirect mechanism that points towards interference with one or more cellular targets involved with the viral life cycle, thus suggesting its potential effect against other viruses [25]. Although **5a** has previously been found to be inactive against the replication of adenovirus and herpes viruses [25], early findings obtained in our group for its potential in inhibiting DENV serotype 2 replication in cell systems (unpublished data) prompted us to prepare a few additional analogues of this compound, and to evaluate them against other *flaviviruses*, including ZIKV. In particular, our early findings in the case of DENV serotype 2 suggest **5a** as an inhibitor of autophagy and micropinocytosis mechanisms, which are relevant to DENV and other *flaviviruses* [26,27]. This inhibitory effect would likely affect the replication of other *flaviviruses*, which enter host cells via multiple pathways, including micropinocytosis, as has been demonstrated for ZIKV infections in glioblastoma cells [28].

### 2.2. Synthetic Chemistry

**Scheme 1:**
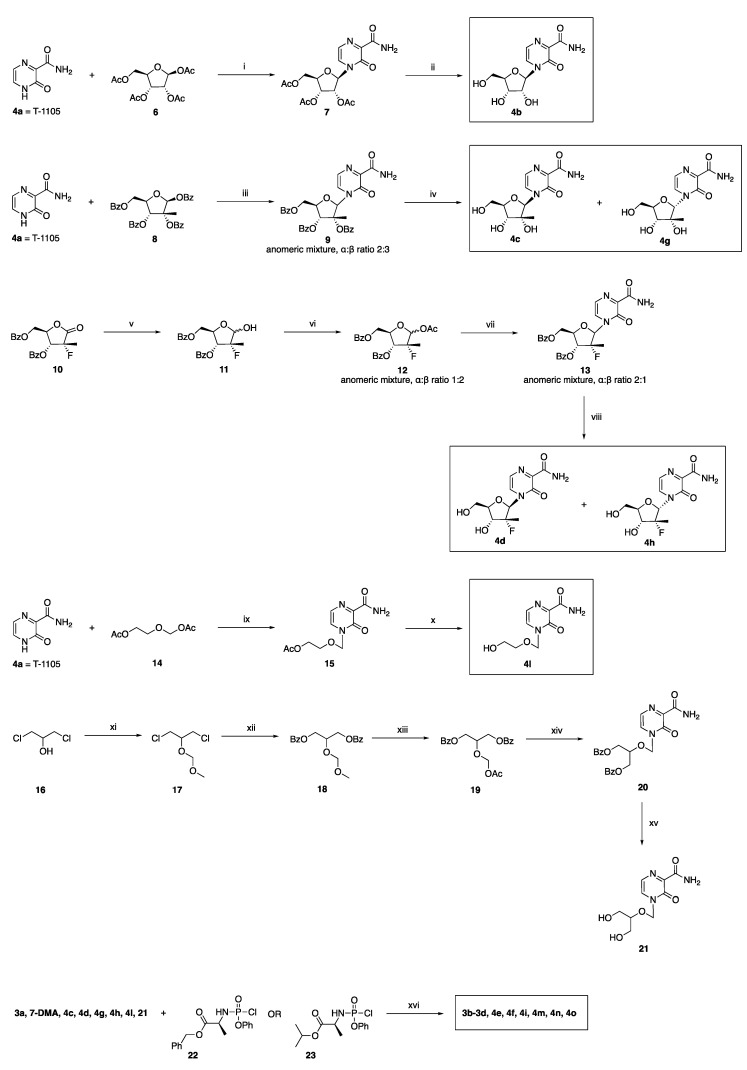
Preparation of nucleoside analogues **3b**–**3d** and **4b**–**4o**. Reagents and conditions: (i) a. BSA, MeCN, reflux, 1h; b. TMSOTf, MeCN, r.t., 2 h (66%); (ii) 25% MeONa/MeOH, 0 °C, 30 min (75%); (iii) a. BSA, MeCN, reflux, 1 h; b. SnCl_4_, r.t., 36 h (82%); (iv) 7N NH_3_/MeOH, r.t., o.n. (39% **4c**, 25% **4g**); (v) LiAlH(*t*BuO)_3_, an. THF, −20 °C, 3 h; (vi) Ac_2_O, DMAP, −20 °C, 2 h (99% over two steps); (vii) a. **4a**, BSA, MeCN, reflux, 1h; b. SnCl_4_, 50 °C, 24 h (75%); (viii) 7N NH_3_/MeOH, r.t., o.n. (25% **4d**, 46% **4h**); (ix) a. BSA, MeCN, reflux, 1 h; b. SnCl_4_, r.t., o.n. (81%); (x) 25% MeONa/MeOH, 0 °C, 30 min (78%); (xi) dimethoxymethane, P_2_O_5_, CHCl_3_, 0 °C to r.t., 18 h (76%); (xii) PhCOONa, 15-Crown-5, DMF, reflux, 18 h (99%); (xiii) BF_3_*Et_2_O, Ac_2_O, 0 °C, 2 h (98%); (xiv) a. **4a**, BSA, MeCN, reflux, 1 h; b. SnCl_4_, r.t., o.n. (80%); (xv) 7N NH_3_/MeOH, r.t., o.n. (69%); (xvi) NMI, THF, Pyr, r.t., o.n. (36–57%).

The preparation of **1a**–**1o** and **2a**–**2m** has recently been reported by our group [17,19], while **3a** and **4a** are commercially available. T-1105 analogues **4b**–**4o** were prepared according to the synthetic routes summarised in Scheme 1, which also shows the preparation of novel aryloxyphosphoramidates **3b**–**3d**.

Preparation of **4b**, the ribofuranosyl form of **4a**, has been previously described [29], and was achieved by reacting commercially available **4a** with tetra-*O*-acetyl-β-d-ribofuranose **6**, according to a Vorbrüggen coupling procedure [30], which yields acetylated nucleoside **7**, then deprotected in a methanolic sodium methoxide solution. Synthesis of both anomers of the 2′-C-methylated derivative, **4c** and **4g**, has also been previously described [31], and was carried out by coupling in Vorbrüggen conditions **4a** with commercial 2-C-methyl-β-d-ribofuranose tetrabenzoate **8**. The coupling step of the reaction had to be performed at room temperature, as attempts under reflux conditions or at 50 °C led to the selective formation of the α-anomer only of protected product **9**. Performing the coupling step at room temperature allowed the obtainment of both anomers of **9**, with an α/β ratio of 2/3. The two anomers were separated after removal of the benzoyl protecting groups in **9**, carried out in methanolic ammonia, through a reverse-phase column chromatography purification of the final reaction mixture containing **4c** and **4g**. 2′-Deoxy-2′-α-fluoro-2′-β-C-methyl analogues **4d** and **4h** have not been previously described. Their preparation began with the reduction in and subsequent acetylation of commercially available lactone **10**, to obtain the acetylated corresponding alcohol **12** in a 1/2 α/β anomeric mixture, following a reported procedure [32]. Coupling of **12** with pseudobase **4a** was once again achieved in Vorbrüggen conditions. After chromatographic purification, a 1/1 α/β anomeric mixture was isolated for **13**. The two anomers **4d** and **4h** were separated after removal of the benzoyl protecting groups, which was carried out in methanolic ammonia, through flash column chromatography purification. Additionally, the preparation of novel acyclic analogues **4l** and **21** has not been previously reported. Compound **4l** was obtained by coupling **4a** with (2-acetoxyethoxy)methyl acetate **14** in Vorbrüggen conditions, as previously reported for the synthesis of various acyclic nucleoside analogues [33], followed by deprotection of the residual acetyl group in methanolic ammonia. Preparation of **21** was achieved following the same approach, by coupling **4a** with 2-(acetoxymethoxy)propane-1,3-diyl dibenzoate **19** to obtain the protected final intermediate **20**, which was treated in methanolic ammonia to achieve the removal of the benzoyl groups in **21**. Then, **19** was prepared following a reported three-step synthetic procedure [34], starting from 1,3-dichloropropan-2-ol **16**. Finally, novel aryloxyphosphoramide prodrugs **3b**–**3d**, **4e**–**4f**, **4i**, **4m**, **4n**, and **4o** were prepared following a coupling reaction between the corresponding nucleoside analogues **3a**, commercial 7-DMA, **4c**, **4d**, **4g**, **4h**, **4l**, and **21** and the appropriate aryl amino acid phosphorochloridate **22** or **23**, in the presence of *N*-methylimidazole (NMI), according to previously validated procedures for the synthesis of ProTide pronucleotides [35]. Then, **22** and **23** were prepared starting from the corresponding ester of *L*-alanine (either isopropryl or benzyl), and reacting it with phenyl dichlorophosphate, as extensively reported in the literature [35].

**Scheme 2:**
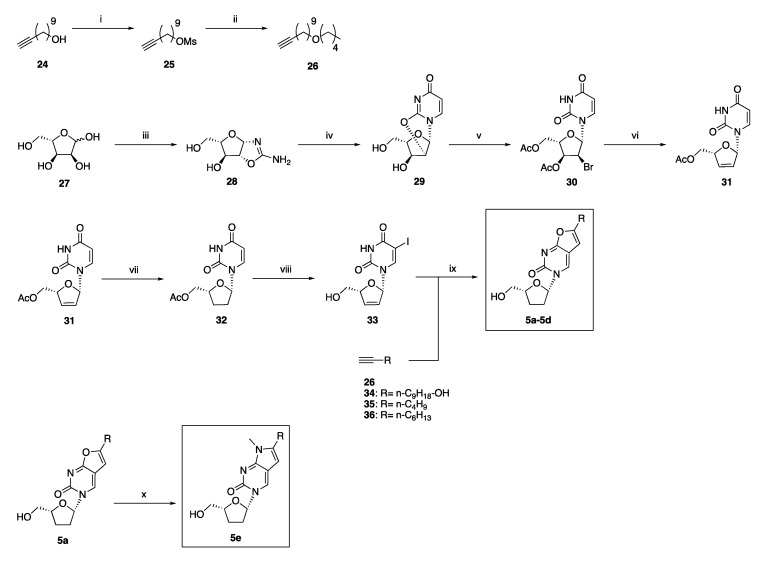
Preparation of nucleoside analogues **5a**–**5e**. Reagents and conditions: (i) MsCl, Et3N, THF, rt, 18 h (97%); (ii) *n*-pentanol, NaH, THF, 70 °C, 24 h (71%); (iii) NC-NH_2_, NH_4_OH, MeOH, rt, 3 days (65%); (iv) methyl propiolate, EtOH, reflux, 3 h (70%); (v) AcBr, MeCN, reflux, 2 h (72%); (vi) Zn, glacial AcOH, EtOH, reflux, 2 h (55%); (vii) H_2_, Pd/C, MeOH, rt, 18 h (95%); (viii) a. I_2_, CAN, MECN, reflux, 6 h, b. MeONa, MeOH, rt, 18 h (56% over two steps); (ix) Pd(PPh_3_)_4_, CuI, DIPEA, DMF, rt, 18 h, then addition of Et_3_N, CuI, 80 °C, 8 h (28–55%); (x) a. MeNH_2_, MeOH, 60 °C, 6 h, b. *p*-TsCl, 1,4-dioxane, PhMe, reflux, 1 h (25% over two steps).

Scheme 2 describes the preparation of the final family of tested compounds, **5a**–**5e**.

Preparation of **5a** and **5d** has been previously described [25], and the reported method was followed also to obtain **5b** and **5c**. Briefly, commercial carbohydrate **27** was reacted with cyanamide to afford the bicyclic oxazoline **28**, which was treated with methyl propiolate in methanol to provide the 2,2′-anhydro-nucleoside **29**. Following a bromoacetylation reaction with acetyl bromide in acetonitrile, **29** was converted into nucleoside **30**, which was subjected to a reductive β-elimination with activated zinc powder in the presence of glacial acetic acid, to afford 5′-*O*-acetyl-2′,3′-didehydro-2′,3′-dideoxyuridine **31**. The dideoxy nucleoside **32** was then obtained upon catalytic hydrogenation of **31**, while preparation of *L*-5-iodo-2′,3′-dideoxyuridine **33** was achieved after iodination at the C5-position of the nucleobase with iodine in the presence of ammonium cerium(IV) nitrate (CAN), followed by deacetylation with sodium methoxide. A palladium-catalysed Sonogashira coupling of **33** with acetylenes **26** and **34**–**36** provided the corresponding 5-alkynyl nucleosides, which, after addition of copper (I) iodide in situ with heating, afforded the desired furanopyrimidine products **5a**–**d**, following an intramolecular cyclisation. While **34**–**36** are commercially available, **26** was prepared in two steps as previously described [29], starting from undec-10-yn-1-ol **24**. Preparation of the *N*-methyl-pyrrolo-pyrimidin-2-one analogue **5e** has also been previously described [36], and was obtained from **5a**, which was treated with methylamine in methanol while heating, in order to achieve the opening of the fused furane ring by the amine. This was then followed by refluxing the reaction in a higher-boiling solvent mixture after treatment with *p*-TsCl, to promote a dehydration and cyclisation of the system, with the formation of the target pyrro analogue **5e**.

### 2.3. Antiviral Studies

The selected compounds were first evaluated at a fixed 10 μM concentration for their potential inhibition of ZIKV-induced cytopathic effect in a MTS based TCID_50_ assay [37], using cell lines which are physiologically relevant for ZIKV infection, including HUH7 hepatoma cells [38], dbtrg cells [28], and A549 cells, which support Zika virus infection in vitro [39]. The cell lines used were chosen to reflect the pathogenesis of ZIKV in liver (HUH7) and CNS tissues (dbtrg), in addition to a cell line where the ZIKV strain originally isolated in Uganda in 1947 grows and forms plaques particularly well (A549). The compounds’ cytotoxicity was also evaluated in the same assay. Ribavirin was used as a positive control [40].

The results of this preliminary assay are summarised in Table 2.

From this evaluation, most of the structural families tested did not show a protective effect against ZIKV-induced cytotoxicity in the cell lines considered. While analogues within the series of compounds **2a**–**2m** and **4a**–**4o** did not show antiviral activity nor relevant cytotoxic effects, several analogues of the first structural scaffold (**1a**–**1o**) were found to show some significant cytotoxicity in this assay. Surprisingly and in contrast with what had been previously reported [19], **2a** did not show a protective effect against ZIKV-induced cytotoxicity in the cell lines used for our assays. Compounds **3a**, **3b**–**3d**, **5a**, **5d**, and **5e** reduced ZIKV-induced cytopathic effect in at least one cell line, showing a significant increase in cell viability in comparison with untreated, infected cells (control-ZIKV), with % values indicating antiviral EC_50_s in the low micromolar range. The activity observed for **3a** is in line with previous literature, which highlights the anti-ZIKV properties of 2′-CMA and 7-DMA [21,22]: as **3b**–**3d** are novel ProTide prodrugs of either one of these two nucleoside analogues, their observed anti-ZIKV effect suggests that application of this pronucleotide technology to 2′-CMA and 7-DMA is associated with an enhanced antiviral effect in comparison with the parent nucleosides. The effect observed for **5a**, which is a known indirect antiviral agent [25], and to a lesser extent for **5d** and **5e**, suggests the very promising potential to explore novel host pathways for the interference with ZIKV life cycle. Due to their protective effect against ZIKV in multiple cell lines, dose–response curves were determined for novel prodrugs **3b**–**3d** and **5a** in the HUH7 cell line, as reported in Table 3. Sofosbuvir was included for this evaluation as a positive control. From this analysis, **3c** and **3d** emerged as the best antiviral hits found in this study, and their antiviral dose–response curves were also determined in the U251 cell line [41], which is an astrocytoma cell line susceptible to ZIKV infection, commonly used as an in vitro model for ZIKV CNS infection.

From this evaluation, **3c** in particular emerges as a promising hit molecule for further development, as it shows a low micromolar antiviral effect in both cell lines, and it is associated with the highest selectivity index found in U251 cells, while it retains EC_50_ values comparable to the ones found for sofosbuvir in our assay.

**Figure 3:**
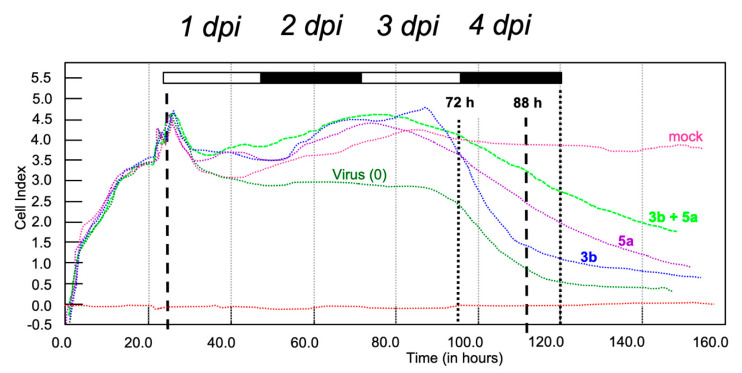
The effect of **3b** (blue), **5a** (purple), and a combination of both (light green) was tested at a concentration of 20 μm against ZIKV in A-549 cells plated in a 16 well CIM-Plate 16 using the xCELLigence RTCA DP Instrument. No compound (dark green; 0) and mock (no virus, pink) were used as controls. Dpi: days postinfection.

Finally, we also conducted a preliminary investigation to explore the possibility of combining the effects of an anti-ZIKV nucleoside analogue, which most likely targets the viral polymerase, and of an agent that likely targets a host pathway. For this evaluation, we tested the effect of the combination of **3b** and **5a** in the A-459 cell line, using an assay that measures cell adherence as an indication of cell viability [42]. Our findings are summarised in Figure 3.

In our assay, cells showed an increase in impedance up to a cell index (CI) of 3.5 in the first 24 h after plating, representing increasing cell–cell contacts and adherence to the well bottom. At this point, the medium was removed (spike; first dotted line in Figure 3) and antiviral compounds were added for a 30-min pretreatment, then ZIKV was added. At the end of the first day of infection (1 day postinfection (dpi)), CI varied, with CI values higher than mock in the combination (light green) and with **5a** alone. Virus without compounds (dark green) showed a beginning of loss of impedance, reflecting ZIKV cytopathological effect of cells rounding, with loss of adherence and loss of electrical resistance (impedance). Up to 2 days of infection, the CI values increased, except in the virus control, whereas at 3 dpi (72 h), a significant separation of impedance was observed: the combination curve is similar to the CI value of the uninfected control (mock), indicating full protection. The wells treated with single compounds show less protection with lower CI values. CI value for uninfected cells (mock) remains at the same level until the end of the experiment. All other CI values drop up to 88/96 h postinfection, when the dropping of CI value for the virus only control flattens out, but does not reach a plateau. Relative efficacy of compounds can be determined at any point between 72 and 96 h postinfection. From 72 h postinfection, the effect of the combination of **3b** and **5a** was more effective than the individual compounds. With the known mechanism of action of the active form of **3b** being viral polymerase inhibition, and the mechanism of action of **5a** being most likely interference with a host pathway, and with early data found in our lab indicating autophagy inhibition (unpublished results), the observed increased efficacy suggesting synergy. In addition, the calculated EC_50_ of the 1:1 combination of **3b** and **5a** in HUH7 cells is 1 ± 0.2 μM, supporting the effects observed on cell impedance/cell-adherence using thexCELLigence RTCA DP instrument.

In conclusion, antiviral evaluation in multiple cell lines of a focused internal library of small-molecule compounds led to the identification of multiple inhibitors of ZIKV-induced cytophatic effect. While we identified three novel ProTide pronucleotides of 2′-CMA and 7-DMA, **3b**, **3c**, and **3d**, as potential leads for the treatment of ZIKV infections, we also found a highly interesting antiviral activity for a l-dideoxy bicyclic pyrimidine nucleoside analogue, **5a**, which had been previously reported to potently inhibit the replication of vaccinia and measles viruses in cells, by interfering with a host pathway. In addition to confirming the anti-ZIKV activity of **5a** on its own, we also observed a clear synergistic effect for its combination with our ProTide pronucleotide **3b**, potentially paving the way for a combination approach for the treatment of ZIKV infection, and also suggesting a novel, host-directed approach for the development of new and improved antiflaviviral strategies. Further exploration of both structural scaffolds and of the synergistic effect observed, along with the elucidation of the precise mechanism of the anti-ZIKV activity of **5a**, are currently ongoing.

## 3. Materials and Methods

### 3.1. Synthetic Chemistry

#### 3.1.1. General Methodologies

All solvents and reagents were used as obtained from commercial sources unless otherwise indicated. All solvents used for chromatography were HPLC grade from Fisher Scientific (UK). All reactions were performed under a nitrogen atmosphere. ^1^H, ^13^C, ^19^F, and ^31^P-NMR spectra were recorded with a Bruker Avance III HD spectrometer operating at 500 MHz for ^1^H and 125 MHz for ^13^C, with Me_4_Si as internal standard. Deuterated chloroform was used as the solvent for NMR experiments, unless otherwise stated. ^1^H chemical shift values (δ) are referenced to the residual non-deuterated components of the NMR solvents (δ = 7.26 ppm for CHCl_3_, etc.). The ^13^C chemical shifts (δ) are referenced to CDCl_3_ (central peak, δ = 77.0 ppm). TLC was performed on silica gel 60 F254 plastic sheets. Normal-phase or reverse-phase automated flash column chromatography was performed using a Biotage Isolera system. UPLC-MS analysis was conducted on a Waters UPLC system with both Diode Array detection and Electrospray (+′ve and −′ve ion) MS detection. The stationary phase was a Waters Acquity UPLC BEH C18 1.7 um 2.1 × 50 mm column. The mobile phase was LC-MS grade H_2_O containing 0.1% formic acid (A) and LC-MS grade MeCN containing 0.1% formic acid (B). Column temperature: 40 °C. Sample diluent: MeCN or H_2_O. Sample concentration 1 µg/mL. Injection volume 2 µL. Four alternative methods were used:

- Linear gradient standard method (A): 90% A (0.1 min); 90–0% A (2.5 min); 0% A (0.3 min); 90% A (0.1 min); flow rate 0.5 mL/min.
- Linear gradient standard method (B): 90% A (0.1 min); 90–0% A (2.1 min); 0% A (0.8 min); 90% A (0.1 min); flow rate 0.5 mL/min.
- Linear gradient standard method (C): 90% A (0.1 min); 90–0% A (1.5 min); 0% A (1.4 min); 90% A (0.1 min); flow rate 0.5 mL/min.
- Linear gradient standard method (D): 99% A (0.1 min); 99–80% A (2.1 min); 0% A (0.8 min); 99% A (0.1 min); flow rate 0.5 mL/min.

High resolution mass spectra (HRMS) were measured in positive mode electrospray ionisation (ES+). All compounds tested in biological assays were >95% pure. Purity of intermediates was >90%, unless otherwise stated. All known intermediates and known compounds were either purchased (**3a**, **7**–**DMA**, **4a**), or prepared according to literature procedures (**1a**–**1o**, **2a**–**2m**, **4b**, **4c**, **4g**, **5a**, **5d**, **5e**), with their characterisation data in line with previously reported data. Details for the preparation and full characterisation of novel compounds are given below.

#### 3.1.2. General Method for the Preparation of Novel ProTides **3b**–**3d**, **4e**, **4f**, **4i**, **4m**–**4o**

The appropriate nucleoside **3a**, **7**–**DMA**, **4c**, **4d**, **4g**, **4h**, **4l**, or **21** (1 eq., 0.5 mmol) was suspended in a mixture of anhydrous tetrahydrofuran/pyridine (1:2 *v*/*v*, 6 mL) at room temperature under a nitrogen atmosphere. 1-Methylimidazole (6 eq., 3 mmol) was then added dropwise and the mixture was stirred for 20 min. A solution of **23** or **24** (1.5 eq., 0.75 mmol) in anhydrous tetrahydrofuran (4 mL) was then added dropwise and the reaction was stirred at room temperature overnight. The reaction was concentrated under vacuum and the crude residue was purified twice by automated flash column chromatography on silica gel (ethyl acetate to ethyl acetate/methanol 90:10 *v*/*v*) to afford the pure title compounds as 1:1 diastereomeric mixtures.

##### 2′-C-Methyladenosine 5′-*O*-[phenyl-(benzyloxy-l-alaninyl)] Phosphate **3b**

Obtained in 36% yield as a white solid. ^1^H-NMR (CD_3_OD), δ: 8.24–8.22 (m, 2H), 7.35–7.17 (m, 10H), 6.13, 6.11 (2s, 1H), 5.16–5.02 (m, 2H), 4.57–4.44 (m, 2H), 4.27–4.18 (m, 2H), 4.07–3.99 (m, 1H), 1.35–1.32 (m, 3H), 0.96, 0.93 (2s, 3H). ^13^C-NMR (CD_3_OD), δ: 174.80, 174.61, 157.42, 157.41, 154.02, 153.99, 152.19, 152.13, 150.44, 150.37, 140.73, 140.46, 137.24, 137.19, 130.81, 130.78, 129.56, 129.53, 129.35, 129.29, 129.24, 129.13, 128.27, 128.00, 126.15, 121.45, 121.44, 121.42, 121.40, 120.38, 93.37, 93.08, 82.26, 82.06, 80.02, 79.94, 74.46, 73.93, 67.95, 67.89, 67.31, 66.39, 51.77, 51.60, 20.51, 20.26, 20.16. ^31^P-NMR (202 MHz, CD_3_OD), δ: 4.01, 3.75. MS [ESI, *m*/*z*]: 621.2 [M + Na]. HRMS calculated for C_27_H_32_N_6_O_8_P^+^: 599.2019; found 599.2037.

##### 7-Deaza-2′-C-methyladenosine 5′-*O*-[phenyl-(benzyloxy-l-alaninyl)] Phosphate **3c**

Obtained in 40% yield as a white solid. ^1^H-NMR (CDCl_3_), δ: 8.28 (s, 1H), 7.39–7.28 (m, 7H), 7.24–7.22 (m, 2H), 7.15–7.03 (m, 2H), 6.39–6.29 (m, 2H), 5.68 (bs, 1H), 5.62 (bs, 1H), 5.38, 5.31 (2s, 2H), 4.54–4.42 (m, 2H), 4.23–4.21 (m, 1H), 4.14–4.01 (m, 2H), 3.45–3.39, 3.06–2.98 (2m, 1H), 1.37–1.31 (m, 3H), 0.82 (s, 3H). ^13^C-NMR (CDCl_3_), δ: 176.60, 173.48, 173.42, 156.57, 156.55, 150.66, 150.58, 149.68, 149.55, 135.19, 135.17, 129.79, 129.78, 128.63, 128.62, 128.49, 128.47, 128.20, 128.16, 125.13, 125.03, 122.17, 122.00, 120.16, 120.09, 99.21, 99.15, 91.44, 91.26, 81.13, 81.00, 78.97, 74.11, 73.79, 67.28, 65.92, 52.84, 50.44, 50.31, 29.70, 20.79, 20.05. ^31^P-NMR (202 MHz, CDCl_3_), δ: 3.21, 2.69. UPLC-MS (Method B): R_t_ 1.68 min, MS [ESI, *m*/*z*]: 598.3 [M + H]. HRMS calculated for C_28_H_33_N_5_O_8_P^+^: 598.2061; found 598.2073.

##### 7-Deaza-2′-C-methyladenosine 5′-*O*-[phenyl-(isopropoxy-l-alaninyl)] Phosphate **3d**

Obtained in 46% yield as a colourless waxy solid. ^1^H-NMR (CDCl_3_), δ: 8.19 (s, 1H), 7.22–7.13 (m, 4H), 7.05–6.93 (m, 2H), 6.32–6.22 (m, 2H), 5.81 (bs, 1H), 5.77 (bs, 1H), 4.93–4.83 (m, 1H), 4.62–4.32 (m, 3H), 4.18–4.12 (m, 1H), 3.98–3.89 (m, 2H), 1.26, 1.25 (2 s, 3H), 1.12–1.04 (m, 6H,), 0.71, 0.69 (2s, 3H). ^13^C-NMR (CDCl_3_), δ: 173.24, 173.19, 173.14, 156.91, 156.89, 151.55, 151.48, 150.71, 150.66, 149.69, 149.58, 129.75, 125.04, 124.97, 121.84, 121.71, 120.13, 120.11, 120.09, 120.07, 103.43, 103.35, 99.16, 91.43, 91.27, 80.70, 80.64, 73.93, 73.58, 69.34, 69.28, 65.89, 65.85, 65.26, 65.23, 50.48, 50.36, 21.65, 21.57, 20.93, 20.89, 20.84, 20.80, 19.89, 19.86. ^31^P-NMR (202 MHz, CDCl_3_), δ: 3.38, 3.11. MS [ESI, *m*/*z*]: 550.2 [M + H], 572.2 [M + Na]. HRMS calculated for C_24_H_33_N_5_O_8_P^+^: 550.2061; found 550.2049.

##### 4-(2′-C-Methyl-β-d-ribofuranosyl)-3-oxo-3,4-dihydropyrazine-2-carboxamide 5′-*O*-[phenyl-(benzyloxy-l-alaninyl)] Phosphate **4e**

Obtained in 48% yield as a pale yellow waxy solid. ^1^H-NMR (CDCl_3_), δ: 9.04 (bs, 1H), 7.86 (bs, 1H), 7.72–7.65 (m, 1H), 7.38–7.31 (m, 8H), 7.24–7.15 (m, 3H), 6.28–6.19 (m, 2H), 5.16, 5.15 (2s, 2H), 4.53–4.46 (m, 1H), 4.40–4.35 (m, 1H), 4.23–4.19 (m, 1H), 4.10–4.03 (m, 1H), 3.87 (d, *J* = 7.5 Hz, 1H), 3.75 (bs, 2H), 1.42–1.38 (m, 3H), 0.97, 0.96 (2s, 3H). ^13^C-NMR (CDCl_3_), δ: 175.66, 168.82, 164.78, 163.92, 157.46, 150.42, 143.64, 135.04, 129.93, 128.70, 128.62, 128.61, 128.24, 128.14, 127.42, 127.21, 125.43, 125.38, 119.98, 119.89, 99.98, 92.40, 90.00, 89.69, 81.63, 80.03, 75.27, 73.18, 72.92, 67.49, 64.49, 50.54, 50.41, 20.82, 20.78, 20.63, 20.61.^31^P-NMR (202 MHz, CDCl_3_), δ: 3.60, 3.19. UPLC-MS (Method B): R_t_ 1.71 min, MS [ESI, *m*/*z*]: 625.2 [M + Na]. HRMS calculated for C_27_H_32_N_4_O_10_P^+^: 603.1851; found 603.1839.

##### 4-[(2’-*R*)-2’-C-Deoxy-2’-C-fluoro-2′-C-methyl-β-d-ribofuranosyl]-3-oxo-3,4-dihydropyrazine-2-carboxamide 5′-*O*-[phenyl-(benzyloxy-l-alaninyl)] Phosphate **4f**

Obtained in 55% yield as a white glassy solid. ^1^H-NMR (CDCl_3_), δ: 9.17–9.12 (bs, 1H), 7.75–7.64 (m, 1H), 7.59–7.50 (m, 1H), 7.40–7.31 (m, 7H), 7.25–7.18 (m, 3H), 6.52, 6.47 (2d, *J_F_* = 18.2 Hz, 1H), 6.27 (bs, 1H), 5.18–5.16 (m, 2H), 4.63–4.55 (m, 1H), 4.51–4.46 (m, 1H), 4.24–4.07 (m, 3H), 4.01, 3.96 (2d, *J* = 8.9 Hz, 1H), 3.85–3.74 (m, 1H), 1.45–1.42 (m, 3H), 1.35–1.30 (m, 3H). ^13^C-NMR (CDCl_3_), δ: 173.87, 173.52, 163.21, 163.12, 155.40, 155.31, 150.43, 150.38, 143.94, 143.74, 134.98, 134.93, 130.05, 130.01, 128.72, 128.60, 126.89, 126.43, 125.58, 125.36, 120.61, 120.57, 119.69, 119.08, 101.18, 101.11, 99.70, 99.63, 89.38, 89.08, 80.34, 80.09, 70.83, 70.71, 67.57, 67.50, 63.57, 63.42, 50.61, 50.39, 30.19, 29.71, 20.82, 20.67, 16.59, 16.39. ^19^F-NMR (470 MHz, CDCl_3_), δ: −163.21, −163.32. ^31^P-NMR (202 MHz, CDCl_3_), δ: 4.26, 3.52. UPLC-MS (Method B): R_t_ 1.80 min (diast. 1), 1.82 min (diast. 2), MS [ESI, *m*/*z*]: 605.2 [M + H]. HRMS calculated for C_27_H_31_FN_4_O_9_P^+^: 605.1807; found 605.1831.

##### 4-[(2’-*R*)-2’-C-Deoxy-2’-C-fluoro-2′-C-methyl-α -d-ribofuranosyl]-3-oxo-3,4-dihydropyrazine-2-carboxamide 5′-*O*-[phenyl-(benzyloxy-l-alaninyl)] Phosphate **4i**

Obtained in 57% yield as a white glassy solid. ^1^H-NMR (CDCl_3_), δ: 9.04, 9.02 (2d, *J* = 3.1 Hz, 1H), 7.72 (app. d, *J* = 4.2 Hz, 1H), 7.50–7.48 (m, 1H), 7.38–7.30 (m, 7 H), 7.24–7.15 (m, 3H), 6.38, 6.27 (2d, *J_F_* = 19.2 Hz, 1H), 6.28–6.14 (m, 1H), 5.19 (d, *J* = 5.7 Hz, 1H), 5.14 (s, 1H), 4.42–4.25 (m, 4H), 4.23–4.03 (m, 2H), 3.92–3.80 (m, 1H), 1.55, 1.42 (2d, *J_F_* = 22.5 Hz, 3H), 1.41–1.36 (m, 3H). ^13^C-NMR (CDCl_3_), δ: 173.87, 173.40, 163.10, 155.55, 150.45, 150.33, 143.69, 143.58, 135.14, 135.06, 129.92, 129.86, 128.71, 128.69, 128.62, 128.55, 128.23, 127.93, 125.58, 125.35, 124.40, 124.33, 120.61, 120.16, 100.07, 99.93, 98.54, 98.40, 87.46, 87.32, 81.78, 81.66, 73.70, 73.56, 67.49, 67.39, 64.85, 64.25, 50.49, 50.30, 20.83, 20.62, 17.74, 17.54. ^19^F-NMR (470 MHz, CDCl_3_), δ: −176.77, −176.84. ^31^P-NMR (202 MHz, CDCl_3_), δ: 3.68, 2.94. UPLC-MS (Method B): R_t_ 1.77 min, MS [ESI, *m*/*z*]: 605.2 [M + H]. HRMS calculated for C_27_H_31_FN_4_O_9_P^+^: 605.1807; found 605.1795.

##### Benzyl ((2-((3-carbamoyl-2-oxopyrazin-1(2*H*)-yl)methoxy)ethoxy)(phenoxy)phosphoryl)-l-alaninate **4m**

Obtained in 51% yield as a pale yellow oil. ^1^H-NMR (CDCl_3_), δ: 9.07 (bs, 1H), 7.69, 7.68 (2d, *J* = 4.1 Hz, 1H), 7.53, 7.48 (2d, *J* = 4.1 Hz, 1H), 7.37–7.29 (m, 7H), 7.21–7.14 (m, 3H), 6.24 (bs, 1H), 5.40 (d, *J* = 2.5 Hz, 1H), 5.35 (d, *J* = 5.3 Hz, 1H), 5.16 (s, 1H), 5.13 (s, 1H), 4.27–4.16 (m, 2H), 4.14–4.02 (m, 1H), 3.86–3.76 (m, 2H), 3.75–3.69 (m, 1H), 1.40, 1.38 (2d, *J* = 7.1 Hz, 3H). ^13^C-NMR (CDCl_3_), δ: 173.48, 173.41, 173.34, 162.98, 155.94, 155.92, 150.66, 150.63, 144.85, 144.81, 135.24, 135.21, 130.01, 129.97, 129.73, 129.71, 128.68, 128.66, 128.58, 128.53, 128.23, 128.19, 125.06, 124.87, 124.86, 120.22, 120.18, 120.12, 120.08, 77.90, 69.72, 69.67, 67.29, 67.27, 65.65, 65.61, 50.41, 50.31, 20.95, 20.91. ^31^P-NMR (202 MHz, CDCl_3_), δ: 2.68, 2.36. UPLC-MS (Method B): R_t_ 1.81 min, MS [ESI, *m*/*z*]: 553.2 [M + Na]. HRMS calculated for C_24_H_28_N_4_O_8_P^+^: 531.1639; found 531.1645.

##### Isopropyl ((2-((3-carbamoyl-2-oxopyrazin-1(2*H*)-yl)methoxy)ethoxy)(phenoxy) phosphoryl)-l-alaninate **4n**

Obtained in 55% yield as an off-white waxy solid. ^1^H-NMR (CDCl_3_), δ: 9.11 (bs, 1H), 7.72 (d, *J* = 4.1 Hz, 1H), 7.57, 7.55 (2d, *J* = 4.1 Hz, 1H), 7.34–7.30 (m, 2H), 7.22–7.14 (m, 3H), 6.03 (bs, 1H), 5.43 (d, *J* = 2.4 Hz, 1H), 5.41 (d, *J* = 4.5 Hz, 1H), 5.02–4.96 (m, 1H), 4.30–4.23 (m, 2H), 4.03–3.92 (m, 1H), 3.88–3.83 (m, 1H), 3.61–3.52 (m, 1H), 1.37–1.34 (2d, *J* = 6.9 Hz, 3H), 1.24–1.20 (m, 6H). ^13^C-NMR (CDCl_3_), δ: 173.51, 173.45, 173.30, 162.89, 155.90, 155.88, 150.59, 150.55, 144.90, 144.85, 130.01, 129.76, 129.74, 125.09, 124.98, 120.23, 120.19, 120.16, 120.12, 77.92, 69.80, 69.75, 69.42, 53.47, 50.40, 50.33, 21.72, 21.64, 21.09. ^31^P-NMR (202 MHz, CDCl_3_), δ: 2.80, 2.55. UPLC-MS (Method B): R_t_ 1.66 min, MS [ESI, *m*/*z*]: 505.2 [M + Na]. HRMS calculated for C_20_H_28_N_4_O_8_P^+^: 483.1639; found 483.1651.

##### Benzyl ((2-((3-carbamoyl-2-oxopyrazin-1(2*H*)-yl)methoxy)-3-hydroxypropoxy)(phenoxy) phosphoryl)-l-alaninate **4o**

Obtained in 54% yield as a pale yellow solid. ^1^H-NMR (CDCl_3_), δ: 9.01 (bs, 1H), 7.65–7.61 (m, 2H), 7.32–7.27 (m, 7H), 7.17–7.10 (m, 3H), 6.60 (bs, 1H), 5.51–5.43 (m, 1H), 5.41–5.32 (m, 1H), 5.12, 5.09 (2s, 2H), 4.23–4.04 (m, 2H), 4.02–3.87 (m, 2H), 3.70-–3.52 (m, 2H), 1.37–1.34 (m, 3H). ^13^C-NMR (CDCl_3_), δ: 173.58, 173.45, 163.58, 155.89, 150.54, 144.13, 135.22, 131.43, 129.76, 129.73, 128.68, 128.64, 128.56, 128.50, 128.21, 128.15, 127.87, 125.13, 124.73, 120.27, 120.23, 120.17, 120.14, 120.11, 79.83, 77.76, 67.27, 66.01, 61.17, 61.10, 50.42, 50.30, 20.79, 20.76. ^31^P-NMR (202 MHz, CDCl_3_), δ: 3.34, 2.98. UPLC-MS (Method B): R_t_ 1.74 min, MS [ESI, *m*/*z*]: 583.2 [M + Na]. HRMS calculated for C_25_H_30_N_4_O_9_P^+^: 561.1745; found 561.1763.

#### 3.1.3. Preparation of Novel 2′-Deoxy-2′-α-fluoro-2′-β-C-methyl-ribofuranosyl Analogues **4d** and **4h**

Commercially available **4a** (20 mmol, 1.5 eq.) was suspended in anhydrous MeCN (50 mL) under a nitrogen atmosphere. *N*,*O*-Bis(trimethylsilyl)acetamide (BSA, 40 mmol, 3 eq.) was then added, and the mixture was stirred under reflux for 1 h. The resulting solution was then cooled to room temperature, and acetylated alcohol **12**, obtained in a 1/2 α/β anomeric mixture according to a previously reported procedure [32] (13.5 mmol, 1 eq.), was dissolved in anhydrous MeCN (10 mL) and added to the reaction mixture. Finally, the mixture was cooled in an ice-bath and SnCl_4_ (47 mmol, 3.5 eq.) was slowly added. The reaction was allowed to warm to room temperature and then heated to 50 °C for 24 h. The mixture was then cooled to 0 °C and a cold saturated NaHCO_3_ solution (100 mL) was added slowly while stirring. The residue was then extracted with EtOAc (3 × 100 mL). The combined organic layers were washed with water and brine, dried over Na_2_SO_4_, and concentrated under vacuum. The crude residue was purified twice by automated flash column chromatography on silica gel (ethyl acetate to ethyl acetate/methanol 90:10 *v*/*v*) to afford **13** as a 1/1 α/β anomeric mixture (pale yellow solid, 75%). Compound **13** (1 mmol, 1 eq.) was then added to 7N methanolic ammonia (15 mL), and the mixture was stirred at room temperature overnight. The reaction mixture was then dried under vacuum and the crude residue was purified three times by automated flash column chromatography on silica gel (dichloromethane to dichloromethane/methanol 90:10 *v*/*v*) to afford the pure nucleosides **4d** and **4h**.

##### 4-[(2’-*R*)-2’-C-Deoxy-2’-C-fluoro-2′-C-methyl-β-D-ribofuranosyl]-3-oxo-3,4-dihydropyrazine-2-carboxamide **4d**

Obtained in 25% yield as a pale yellow solid. ^1^H-NMR (DMSO-*d*_6_), δ: 8.24 (d, *J* = 4.4 Hz, 1H), 8.23 (bs, 1H), 7.75 (bs, 1H), 7.56 (d, *J* = 4.4 Hz, 1H), 6.29 (d, *J_F_* = 18.3 Hz, 1H), 5.74 (d, *J* = 6.7 Hz, 1H), 5.43 (app. t, *J* = 4.5 Hz, 1H), 3.97–3.87 (m, 3H), 3.70 (ddd, *J_1_* = 11.7 Hz, *J_2_* = 3.7 Hz, *J_3_* = 1.8 Hz, 1H), 1.21 (d, *J_F_* = 22.5 Hz, 3H). ^13^C-NMR (DMSO-*d*_6_), δ: 164.58, 154.23, 148.66, 126.66, 123.60, 101.59 (d, *J_F_* = 182.3 Hz), 82.65, 70.31 (d, *J_F_* = 17.1 Hz), 58.57, 49.07, 16.87 (d, *J_F_* = 24.9 Hz). ^19^F-NMR (470 MHz, DMSO-*d*_6_), δ: −161.20. UPLC-MS (Method D): R_t_ 1.59 min, MS [ESI, *m*/*z*]: 288.2 [M + H]. HRMS calculated for C_11_H_15_FN_3_O_5_^+^: 288.0990; found 288.0976.

##### 4-[(2’-*R*)-2’-C-Deoxy-2’-C-fluoro-2′-C-methyl-α-D-ribofuranosyl]-3-oxo-3,4-dihydropyrazine-2-carboxamide **4h**

Obtained in 46% yield as a white solid. ^1^H-NMR (DMSO-*d*_6_), δ: 8.21 (bs, 1H), 7.74–7.69 (m, 2H), 7.51 (d, *J* = 4.4 Hz, 1H), 6.38 (d, *J_F_* = 19.2 Hz, 1H), 5.78 (d, *J* = 7.2 Hz, 1H), 4.90 (app. t, *J* = 5.5 Hz, 1H), 4.21–4.18 (m, 1H), 4.06–4.01 (m, 1H), 3.75 (dd, *J_1_* = 12.1 Hz, *J_2_* = 3.5 Hz, 1H), 3.57–3.51 (m, 1H), 1.45 (d, *J_F_* = 22.4 Hz, 3H). ^13^C-NMR (DMSO-*d*_6_), δ: 164.83, 154.18, 148.77, 128.52, 122.75, 99.64 (d, *J_F_* = 191.2 Hz), 87.25 (d, *J_F_* = 14.5 Hz), 84.08, 73.21 (d, *J_F_* = 17.2 Hz), 60.68, 18.23 (d, *J_F_* = 25.1 Hz). ^19^F-NMR (470 MHz, DMSO-*d*_6_), δ: −174.08. UPLC-MS (Method D): R_t_ 0.61 min, MS [ESI, *m*/*z*]: 310.1 [M + Na]. HRMS calculated for C_11_H_15_FN_3_O_5_^+^: 288.0990; found 288.0998.

#### 3.1.4. Preparation of 4-((2-Hydroxyethoxy)methyl)-3-oxo-3,4-dihydropyrazine-2-carboxamide **4l**

Commercially available **4a** (3.6 mmol, 1 eq.) was suspended in anhydrous MeCN (10 mL) under a nitrogen atmosphere. *N*,*O*-Bis(trimethylsilyl)acetamide (BSA, 8.3 mmol, 2.3 eq.) was then added, and the mixture was stirred under reflux for 1 h. The resulting solution was then cooled to room temperature, and commercially available (2-acetoxyethoxy)methyl acetate **14** was added (3.6 mmol, 1 eq.). The mixture was then cooled in an ice-bath and SnCl_4_ (8.3 mmol, 2.3 eq.) was slowly added. The reaction was allowed to warm to room temperature and stirred overnight. The mixture was then cooled to 0 °C, and a cold saturated NaHCO_3_ solution (40 mL) was added slowly while stirring. The residue was then extracted with EtOAc (5 × 50 mL). The combined organic layers were washed with water and brine, dried over Na_2_SO_4_, and concentrated under vacuum. The crude residue was purified by automated flash column chromatography on silica gel (dichloromethane to dichloromethane/methanol 90:10 *v*/*v*) to afford **15** as a yellow oil (81%). Compound **15** (2.5 mmol, 1 eq.) was then dissolved in MeOH (10 mL) and the solution was cooled to 0 °C in an ice-bath. A 25% MeONa solution in MeOH (0.9 mL, 1.5 eq.) was added and the reaction was stirred for 40 min at 0 °C. Glacial acetic acid (3.6 mL) was then added dropwise to the reaction while stirring, and the mixture was dried under vacuum. The crude residue was purified twice by automated flash column chromatography on silica gel (ethyl acetate to ethyl acetate/methanol 85:15 *v*/*v*) to afford the pure title compound **4l** as a pale yellow oil in 78% yield. ^1^H-NMR (CD_3_OD), δ: 7.97 (d, *J* = 3.7 Hz, 1H), 7.70 (d, *J* = 3.7 Hz, 1H), 5.53 (s, 2H), 3.73–3.70 (m, 4H). ^13^C-NMR (CD_3_OD), δ: 164.89, 156.04, 145.05, 132.14, 123.73, 78.32, 71.97, 60.51. UPLC-MS (Method D): R_t_ 0.65 min, MS [ESI, *m*/*z*]: 236.1 [M + Na]. HRMS calculated for C_8_H_12_N_3_O_4_^+^: 214.0822; found 214.0810.

#### 3.1.5. Preparation of 4-(((1,3-Dihydroxypropan-2-yl)oxy)methyl)-3-oxo-3,4-dihydropyrazine-2-carboxamide **21**

Commercially available **4a** (3.6 mmol, 1 eq.) was suspended in anhydrous MeCN (9 mL) under a nitrogen atmosphere. *N*,*O*-Bis(trimethylsilyl)acetamide (BSA, 8.3 mmol, 2.3 eq.) was then added, and the mixture was stirred under reflux for 1 h. The resulting solution was cooled to room temperature, and 2-(acetoxymethoxy)propane-1,3-diyl dibenzoate **19** (3.6 mmol, 1 eq.), prepared following a reported procedure [34], was added dissolved in MeCN (3 mL). The mixture was then cooled in an ice-bath, and SnCl_4_ (8.3 mmol, 2.3 eq.) was slowly added. The reaction was allowed to warm to room temperature and stirred overnight. The mixture was then cooled to 0 °C and a cold saturated NaHCO_3_ solution (40 mL) was added slowly while stirring. The residue was then extracted with EtOAc (3 × 50 mL). The combined organic layers were washed with water and brine, dried over Na_2_SO_4_ and concentrated under vacuum. The crude residue was purified by automated flash column chromatography on silica gel (dichloromethane to dichloromethane/methanol 90:10 *v*/*v*) to afford **20** as an orange oil (80%). Compound **20** (2.8 mmol, 1 eq.) was then added to 7N methanolic ammonia (40 mL), and the mixture was stirred at room temperature overnight. The reaction mixture was then dried under vacuum, and the crude residue was purified by automated flash column chromatography on silica gel (chloroform to chloroform/methanol 80:20 *v*/*v*) to afford the pure title compound **21** as a pale yellow solid in 69% yield. ^1^H-NMR (DMSO-*d*_6_), δ: 8.38 (bs, 1H), 7.96 (d, *J* = 4.1 Hz, 1H), 7.73 (bs, 1H), 7.53 (d, *J* = 4.1 Hz, 1H), 5.46 (s, 2H), 4.69 (t, *J* = 5.5 Hz, 2H), 3.61–3.57 (m, 1H), 3.49–3.45 (m, 2H), 3.40–3.36 (m, 2H). ^13^C-NMR (DMSO-d_6_), δ: 164.48, 155.00, 147.96, 131.63, 123.08, 82.40, 77.34, 61.37. UPLC-MS (Method D): R_t_ 0.36 min, MS [ESI, *m*/*z*]: 266.1 [M + Na]. HRMS calculated for C_9_H_14_N_3_O_5_^+^: 244.0928; found 244.0941.

#### 3.1.6. General Procedure for the Preparation of Novel 3-(2′,3′-Dideoxy-ribo-β-l-furanosyl)-6-substituted-furo[2,3d]pyrimidin-2-(3*H*)one Analogues **5b** and **5c**

l-5-iodo-2′,3′-dideoxyuridine **33** (0.35 mmol, 1 eq.), prepared as previously described [25], was dissolved in anhydrous DMF (2 mL) under a nitrogen atmosphere. The appropriate alkyne **34** or **35** (1.05 mmol, 3 eq.) was then added, followed by tetrakis(triphenylphosphine)palladium (0.03 mmol, 0.1 eq.), CuI (0.07 mmol, 0.2 eq.), and DIPEA (0.7 mmol, 2 eq.). The resulting mixture was stirred at room temperature for 18 h. At this point, further CuI (0.07 mmol, 0.2 eq.) and Et_3_N (2 mL) were added, and the mixture was stirred at 80 °C for 8 h. The residue was dried under vacuum by co-evaporation with EtOAc, and the crude product was purified by automated flash column chromatography on silica gel (dichloromethane to dichloromethane/methanol 92:8 *v*/*v*) to afford the pure target compounds **5b** and **5c**.

##### 3-(2′,3′-Dideoxy-ribo-β-l-furanosyl)-6-(1-nonanol) furo[2,3-d]pyrimidin- 2(3H)-one **5b**

Obtained in 28% yield as an off-white solid. ^1^H-NMR (CDCl_3_), δ: 8.66 (s, 1H), 6.22 (dd, *J_1_* = 4.1 Hz, *J_2_* = 2.5 Hz, 1H), 6.13 (d, *J* = 7.5 Hz, 1H), 4.31 (m, 1H), 4.04−4.01 (m, 1H), 3.79 (1H, dt, J_1_ = 12.1 Hz, J_2_ = 4.2 Hz, 1H), 3.66 (m, 2H), 2.68–2.60 (m, 3H), 2.26–2.21 (m, 1H), 2.17 (t, *J* = 4.5Hz, 1H), 1.98−1.91 (m, 2H), 1.73–1.67 (m, 2H), 1.59–1.55 (m, 2H), 1.41−1.32 (m, 11H). ^13^C-NMR (CDCl_3_), δ: 171.57, 159.75, 155.50, 137.21, 107.64, 99.29, 88.15, 83.55, 61.64, 61,59, 33.33, 32.23, 29.16, 29.10, 28.88, 28.64, 27.54, 26.61, 25.50, 23.31. MS [ESI, *m*/*z*]: 379.2 [M + H]. HRMS calculated for C_20_H_31_N_2_O_5_^+^: 379.2227; found 379.2209.

##### 3-(2′,3′-Dideoxy-ribo-β-l-furanosyl)-6-(n-butil)furo[2,3-d]pyrimidin- 2(3*H*)-one **5c**

Obtained in 33% yield as a pale yellow solid. ^1^H-NMR (CDCl_3_), δ: 8.68 (s, 1H), 6.21 (d, *J* = 4.5 Hz, 1H), 6.14 (s, 1H), 4.31 (m, 1H), 4.16 (d, *J* = 11.5 Hz, 1H), 3.89 (d, *J* = 11.5 Hz, 1H), 2.68−2.61 (m, 3H), 2.33 (bs, 1H), 2.24−2.21 (m, 1H),1.97−1.93 (m, 2H),1.69 (m, 1H), 1.41−1.31 (m, 2H), 1.28(m, 1H), 0.91 (t, *J* = 7.2 Hz, 3H). ^13^C-NMR (CDCl_3_), δ: 171.72, 159.46, 155.02, 135.89, 107.24, 99.08, 88.99, 82.88, 62.85, 33.78, 28.87, 27.94, 23.89, 22.11, 13.70. MS [ESI, *m*/*z*]: 293.1 [M + H]. HRMS calculated for C_15_H_21_N_2_O_4_^+^: 293.1496; found 293.1510.

### 3.2. Antiviral Studies

#### 3.2.1. Antivirals

Lyophilized compounds were solubilized in DMSO (Sigma-Aldrich, St. Louis, MO, USA) to a stock concentration of 10 mM and kept frozen at −20 °C and then were diluted to final assay concentrations. Dissolved compounds were stored at −20 °C and brought to room temperature prior to assay. All compounds were well dissolved in DMSO, and precipitates were not observed upon thawing.

#### 3.2.2. Cell Culture

The human hepatoma cell line HUH7 was a gift of Ralf Bartenschlager, University of Heidelberg. Human glioblastoma cell lines U138 and U251 were originally obtained from the American Tissue Type Culture collection (ATCC) and European Collection of Authenticated Cell Cultures (ECACC)—with the numbers U138 MG—ATCC^®^ HTB16™, U251MG ECACC catalogue number 09063001, respectively. Cells were cultured in Dulbecco’s modified Eagle’s medium (DMEM) with 4.5 mg/mL glucose (Gibco Life Technologies Thermo Fisher, Waltham, MA, USA) supplemented with 10% (*v*/*v*) heat inactivated fetal bovine serum (FBS; Gibco Life Technologies) at 37 °C with 5% CO_2_ (DMEM/FC10).

#### 3.2.3. Virus Culture

ZIKV-U (976 Uganda strain; isolated from a non-human primate in Uganda in 1947; strain form the Stammsammlung (strain bank) of the Institut für Mikrobiologie der Bundeswehr) was grown to high titer on Vero B4 cells, partially purified by centrifugation to remove cell debris and stored frozen at −80 °C. Virus titer was determined by standard plaque assay.

#### 3.2.4. Viability Assay

Cell viability was determined after seven days using CellTiter-Glo^®^ (PROMEGA), (Madison, WI, USA) following the manufacturer’s instructions. All experiments were performed in triplicate. Briefly, cells were seeded into white 96 well plates (Greiner-Bio ONE; Kremsmünster, Austria) at a density of 10^4^ cells per well in a volume of 100 µL DMEM/FC10, then incubated for 16 h. Cells were treated in triplicates with the antiviral compound, prediluted to a range of concentrations, and added in a volume of 50 µL DMEM, then immediately infected with ZIKV-U or DENV2, prediluted and added in a volume of 50 µL DMEM, at a multiplicity of infection of 0.3 to the final antiviral concentration in 200 µL final assay volume. Uninfected/untreated controls were prepared in multiple triplicates in different locations on the 96 well plate. Cells were incubated for 4 days at 37 °C with 5% CO_2_. The reconstituted CellTiter-Glo^®^ reagent was then added to the cells and luminescence was detected within 60 min by the Victor X3 2030 Multilabel Reader (PerkinElmer, Waltham, MA, USA) according to the manufacturer’s instructions. Data were analysed using Microsoft EXCEL and Graphpad PRISM 7.0 software.

#### 3.2.5. Cell Adherence Assay

Cell adherence was determined using the xCELLigence assay in the 16 well format [42]. Briefly, cells were plated at a density of 10^4^ cells per well in a 16 well CIM-Plate 16 for the xCELLigence RTCA DP Instrument (ACEA Biosciences, Inc., San Diego, CA, USA). After plating, cells were observed for one hour to confirm adherence and impedance. The monitoring was then suspended for a 30-min pretreatment with compounds followed by infection with virus, after which monitoring was resumed. The instrument recorded impedance over time in each well. The data shown represent averages of duplicates. Data were analysed using the xCELLigence Real-Time Cell Analyzer (RTCA) software.

## Figures and Tables

**Table 1:** Chemical structures of compounds **1a**–**1o** and **2a**–**2m**, previously reported non-nucleoside antiviral agents, selected for evaluation against ZIKV-induced cytopathic effect in relevant cellular models of the infection.

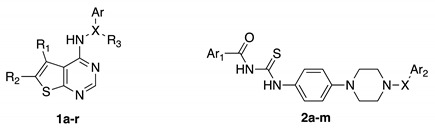							
**Cpd**	**R_1_**	**R_2_**	**X**	**R_3_**	**Ar**	**Ar_1_**	**Ar_2_**
**1a**	C_4_H_8_		N=C	Me	2,5-OH-Ph	-	-
**1b**	C_4_H_8_		N=C	Me	2-OH-Ph	-	-
**1c**	C_4_H_8_		N=C	Me	Pyrazine	-	-
**1d**	H	H	N=C	Me	2,5-OH-Ph	-	-
**1e**	C_4_H_8_		N=C	Me	2-Pyridine	-	-
**1f**	C_3_H_6_		N=C	Me	2-OH-Ph	-	-
**1g**	C_4_H_8_		NHCO	-	2,5-OH-Ph	-	-
**1h**	C_4_H_8_		NHCO	-	2,5-OH-Ph	-	-
**1i**	Me	Me	N=C	Me	2-OH-Ph	-	-
**1l**	C_4_H_4_		N=C	Me	2-OH-Ph	-	-
**1m**	C_4_H_4_		NHCO	-	2-Pyridine	-	-
**1n**	H	H	NHCO	-	2-Pyridine	-	-
**1o**	Me	Cl	NHCO	-	2-Pyridine	-	-
**2a**	-	-	C=O	-	-	2-(3-Cl)-Benzo[*b*]thiophene	2-Thiophene
**2b**	-	-	C=O	-	-	2-Benzo[*b*]thiophene	2-Thiophene
**2c**	-	-	C=O	-	-	2-(3-CF_3_)-Benzo[*b*]thiophene	2-Thiophene
**2d**	-	-	C=O	-	-	4-Me-Ph	2-Thiophene
**2e**	-	-	C=O	-	-	2-(3-Cl)-Benzo[*b*]thiophene	2-Furan
**2f**	-	-	C=O	-	-	2-(3-Cl)-Benzo[*b*]thiophene	Ph
**2g**	-	-	C=O	-	-	Ph	Ph
**2h**	-	-	C=O	-	-	2-(3-Cl)-Benzo[*b*]thiophene	2-Thiazole
**2i**	-	-	C=O	-	-	4-Me-Ph	Ph
**2l**	-	-	SO_2_	-	-	Ph	2-Thiophene
**2m**	-	-	C=O	-	-	Ph	2-Thiazole

**Table 2:** Reduction in ZIKV-induced cytopathic effect (CPE) and cytotoxicity data for the selected compounds at a fixed 10 μM concentration in multiple cell lines ^1^.

Compound	% Cell Viability, ZIKV–Infected HUH7	% Cell Viability, HUH7 (Cytotoxicity)	% Cell Viability, ZIKV–Infected Dbtrg	% Cell Viability, Dbtrg (Cytotoxicity)	% Cell Viability, ZIKV–Infected A459	% Cell Viability, A459 (Cytotoxicity)
**1a**	13.5	23.5	0	15	n.d.	n.d.
**1b**	3.4	14.5	0	17.3	n.d.	n.d.
**1c**	8.8	32	0	18.4	n.d.	n.d.
**1d**	8.3	20	0	24.9	n.d.	n.d.
**1e**	0	11.1	0	14.1	n.d.	n.d.
**1f**	4.9	15.3	0	19.3	n.d.	n.d.
**1g**	6.1	46.2	0	24.3	n.d.	n.d.
**1h**	5.4	26.1	0	21.8	n.d.	n.d.
**1i**	5.7	16.1	0	15.5	n.d.	n.d.
**1l**	12.1	22.0	0	18.2	n.d.	n.d.
**1m**	7.9	25.2	0	12.6	n.d.	n.d.
**1n**	5.1	37.9	0	14.8	n.d.	n.d.
**1o**	5.2	30.6	0	8.9	n.d.	n.d.
**2a**	30.5 ± 15.9	117.9 ± 42.2	n.d.	n.d.	n.d.	n.d.
**2b**	25.5 ± 18.2	107.4 ± 35.9	3.1 ± 3.9	94.9 ± 16.2	n.d.	n.d.
**2c**	0.4 ± 0.7	108.6 ± 55.9	4.1 ± 5.6	88.9 ± 18.5	n.d.	n.d.
**2d**	4.5 ± 6.6	119.5 ± 47.3	2.8 ± 2.8	76.9 ± 25.7	n.d.	n.d.
**2e**	11.2 ± 9.9	117.8 ± 40.9	0.25 ± 0.43	80.5 ± 27.2	n.d.	n.d.
**2f**	3.9 ± 1.9	75.7 ± 54.3	0.8 ± 1.4	76 ± 27.4	n.d.	n.d.
**2g**	9.5 ± 7.1	160.6 ± 50.6	0	109.7 ± 4.8	n.d.	n.d.
**2h**	9.8 ± 6.6	134.9 ± 51.6	2.5 ± 2.2	114.7 ± 4.5	n.d.	n.d.
**2i**	4.5 ± 3.3	106.4 ± 59.1	1.7 ± 1.7	96.7 ± 15.2	n.d.	n.d.
**2l**	1.6 ± 3.6	110.1 ± 63.5	9.2 ± 8.5	91.8 ± 14.5	n.d.	n.d.
**2m**	3.3 ± 2.7	99.9 ± 62.9	11 ± 7.5	104.2 ± 10.7	n.d.	n.d.
**3a** (2′-CMA)	**31.4**	**98.8**	5.2	107.3	**40**	**106.9**
**3b**	**48.2**	**98.2**	10.2	100.5	**33.6**	**103.5**
**3c**	**120.4 ± 80.3**	**137.6 ± 35.6**	**53.3 ± 18.9**	**87.9 ± 18.7**	n.d.	n.d.
**3d**	**101.5**	**107.9**	8.1	115.5	**59.6**	**112.3**
**4a**	0.1	100.6	4.6	97.4	n.d.	n.d.
**4b**	0.1	106.8	4.6	99.2	n.d.	n.d.
**4c**	0.6	118.5	7.3	105.6	n.d.	n.d.
**4d**	0	105.6	0.4	98.7	n.d.	n.d.
**4e**	0	90.8	0	85.9	n.d.	n.d.
**4f**	0.1	97.8	6.6	82.3	n.d.	n.d.
**4g**	0.1	117.5	0	104.9	n.d.	n.d.
**4h**	0	106.5	0.1	99.3	n.d.	n.d.
**4i**	0.2	102.5	4.3	97.8	n.d.	n.d.
**4l**	0	101.3	4.5	98.5	n.d.	n.d.
**4m**	0.2	106.4	1.2	99.6	n.d.	n.d.
**4n**	0.1	104.9	2.2	98	n.d.	n.d.
**4o**	0.1	97.8	6.6	82.3	n.d.	n.d.
**5a**	**50.3**	**100.5**	0	75.7	0	84.8
**5b**	0	110.1	5.7	102.9	8.2	91.6
**5c**	0	104.4	3.9	94.7	0	87.6
**5d**	0.2	104.3	0.4	74.5	**28.6**	**104.7**
**5e**	**30.6**	**55.1**	13.6	52	2.7	81.6
Ribavirin	61.1 ± 26.4	158.7 ± 16.9	25.1 ± 10.1	94.8 ± 21.6	n.d.	n.d.
Control-DMSO	100	100	100	100	100	100
Control-ZIKV	0	6 ± 1.4	0	14.4 ± 4.3	0	0

The most interesting antiviral results obtained are highlighted in bold. ^1^ Reported values are the mean of at least 2 independent experiments, with standard deviations of ±10% of the value quoted unless otherwise stated (mean value ± standard deviations).

**Table 3:** Antiviral EC_50_ and CC_50_ values calculated for selected hits in the HUH7 and U251 cell lines.

Compound	EC_50_^a,c^ (μM), HUH7	CC_50_^b,c^ (μM), HUH7	SI ^d^, HUH7	EC_50_^a,c^ (μM), U251	CC_50_^b,c^ (μM), U251	SI ^d^, U251
**3b**	4 ± 0.2	>20	>5	-	-	-
**3c**	**11.6 ± 1.3**	**211.2 ± 4.6**	**18.2**	**8.2 ± 1.1**	**203.7 ± 16.9**	**24.8**
**3d**	8.3 ± 0.9	128.2 ± 21.1	15.4	8.6 ± 6.5	13.6 ± 1.7	1.6
**5a**	10.2 ± 0.4	>20	>2	-	-	-
Sofosbuvir	5.8 ± 0.8	133.5 ± 13.6	23	7.7 ± 4.5	139.1 ± 26.4	18

^a^ EC_50_ = 50% effective concentration (concentration at which 50% inhibition of virus replication is observed). ^b^ CC_50_ = 50% cytostatic/cytotoxic concentration (concentration at which 50% adverse effect is observed on the host cell). ^c^ The EC_50_ and CC_50_ values are the mean of at least 3 independent experiments, with standard deviations of ±10% of the value quoted unless otherwise stated (mean value ± standard deviations). ^d^ SI = the ratio of CC_50_ to EC_50_.
